# Early Diagnosis of Disseminated *Mycobacterium genavense* Infection

**DOI:** 10.3201/eid1401.070901

**Published:** 2008-02

**Authors:** Victoire de Lastours, Romain Guillemain, Jean-Luc Mainardi, Agnès Aubert, Patrick Chevalier, Agnès Lefort, Isabelle Podglajen

**Affiliations:** * Assistance Publique-Hôpitaux de Paris, Paris, France; † Hôpital Européen Georges Pompidou, Paris, France; ‡ Université Paris-Descartes, Paris, France; § Université Pierre et Marie Curie, Paris, France; ¶ Hôpital Beaujon, Paris, France

**Keywords:** RNA, ribosomal, 16S, Mycobacterium infections, heart, transplant, letter

**To the Editor:** Nontuberculous mycobacteria are environmental organisms that cause life-threatening diseases, particularly in immunocompromised hosts. They are increasingly recognized for causing problems in the management of solid-organ transplant recipients, due to improved diagnostic methods as well as increasing numbers and life expectancy of these patients (*1*). The slow-growing *Mycobacterium genavense* is a ubiquitous nontuberculous mycobacterium; it is reportedly isolated from tap water, pets, and the gastrointestinal tract of healthy humans (*1*,*2*). It was first recognized as a human pathogen in a patient with AIDS but has not yet been found in heart transplant recipients (*3*). We report early diagnosis of disseminated *M. genavense* infection in a heart transplant recipient.

A 37-year-old man was hospitalized in September 2001 for abdominal pain, sweats, and weight loss; he had received a heart transplant 3 years earlier. Immunosuppressive treatment, which began immediately after transplantation, consisted of tacrolimus (5 mg) and mycophenolate mofetil (2 g) daily; concurrent steroid therapy was tapered off over the next 6 months. A computed tomographic (CT) scan showed numerous large lymph nodes in his abdomen (Figure). Endoscopic examinations showed diffuse inflammation of the mucosa of the duodenum, ileum, and colon. Multiple biopsy samples were submitted for histologic analysis, culture, and molecular biological analysis. Immediate 16S rRNA gene amplification that used universal primers (*4*) and sequencing of samples taken directly from the biopsy material led to the identification of *M. genavense*. A 475-bp fragment was sequenced, and 99% homology with the gene of type strain ATCC 51234 (GenBank accession no. X60070) was found. PCR results were positive for 2 of the 4 samples tested. The molecular identification was compatible with the subsequent histologic finding of profound macrophage infiltration without granuloma and the presence of Ziehl-Neelsen–positive bacilli. Five weeks later, the molecular diagnosis was confirmed by blood cultures and cultures of the intestinal mucosa samples (Inno-LiPA Mycobacteria test, version 2, Innogenetics, Courtaboeuf, France). The direct molecular diagnosis of *M. genavense* enabled immediate treatment of the patient with the combination of moxifloxacin, ethambutol, clarithromycin, and amikacin; mycophenolate mofetil was discontinued. Clofazimine was added to the treatment regimen 3 months later, when a control CT scan showed that some of the enlarged mesenteric lymph nodes had increased further. After 5 months, the clinical signs resolved, and after 9 months, the lymph nodes were substantially smaller. CT scan results were within normal limits after 12 months of treatment; only ethambutol and clarithromycin were continued for an additional 6 months. There was no sign of *M. genavense* infection relapse 3 years after the diagnosis had been made.

**Figure:**
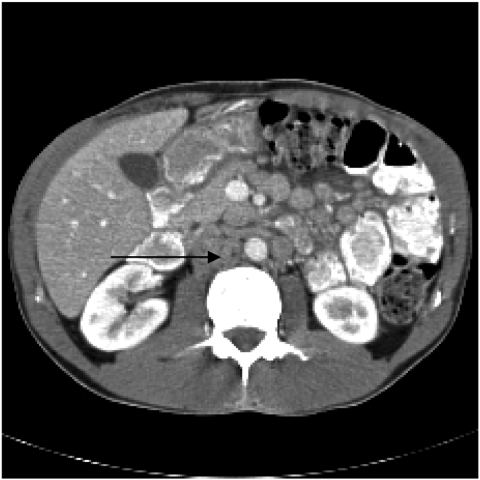
Initial computed tomographic scan of the abdomen, performed after intravenous injection of contrast dye, showing numerous enlarged para-aortic lymph nodes (arrow).

Nontuberculous mycobacteria in persons who have received heart or other solid-organ transplants remain a rare cause of late infectious complications and occur ≈3.5 years after transplantation (*1*,*4*). In the subgroup of heart transplant recipients, skin disease is the most common clinical manifestation, followed by pulmonary and disseminated disease; *M. kansasii, M. avium* complex, and *M. haemophilum* infections are most frequently encountered (*1*,*5*).

*M. genavense* causes up to 12.8% of all nontuberculous mycobacteria infections in AIDS patients; these infections are clinically similar to those caused by the *M. avium* complex (*1*,*6*). *M. genavense* infections occur only rarely in persons other than AIDS patients (as in the present case), but they always occur in immunocompromised persons (*7*,*8*). To date, only 1 case of disseminated infection has been reported in a solid-organ (kidney) transplant recipient; the diagnosis was made by molecular identification in isolates from blood and marrow cultures. That patient died of complications from *M. genavense* infection (*9*). Because *M. genavense* is a fastidious organism, the infections it causes are difficult to diagnose and their frequency is probably underestimated, which may change with increased use of direct molecular biological methods.

Optimal treatment of *M. genavense* infections has not been established (*10*). Experience with *M. genavense* infections in AIDS patients and with other nontuberculous mycobacteria infections in solid-organ transplant recipients suggests that at least 2 antimicrobial drugs should be used for a prolonged period; when possible, immunosuppressive drugs should be concurrently reduced (*1*,*3*,*6*,*10*). Outcome of nontuberculous mycobacteria infections in transplant patients is highly variable (*1*,*5*) but was satisfactory in the present patient, who was treated with quintuple antimicrobial-drug therapy and reduced immunosuppressive therapy.

This case of a disseminated infection due to *M. genavense* in a heart transplant recipient was diagnosed early. Universal 16S rRNA gene sequencing after amplification directly from intestinal biopsy specimens enabled fast diagnosis and appropriate management.
